# Supplementary material for: Hypoxia-Regulated lncRNA USP2-AS1 Drives Head and Neck Squamous Cell Carcinoma Progression
Source: Cells. 2022 Oct 28;11(21):3407. doi: 10.3390/cells11213407 (PMC9655520; doi:10.3390/cells11213407)
Supplement: Supplementary file 1 [file cells-11-03407-s001.zip › cells-1912215-supplementary/suppl materials/1008 suppl figs.pdf]

Fig. S1

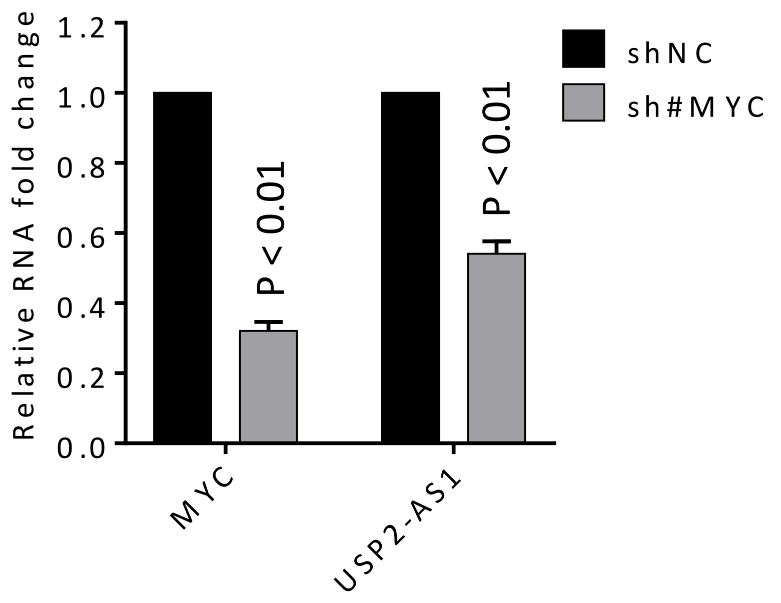

Fig. S1 Silencing MYC inhibits USP2-AS1 in FaDu cells

qRT-PCR analysis revealed that silencing MYC inhibits USP2-AS1 in FaDu cells

Fig. S2

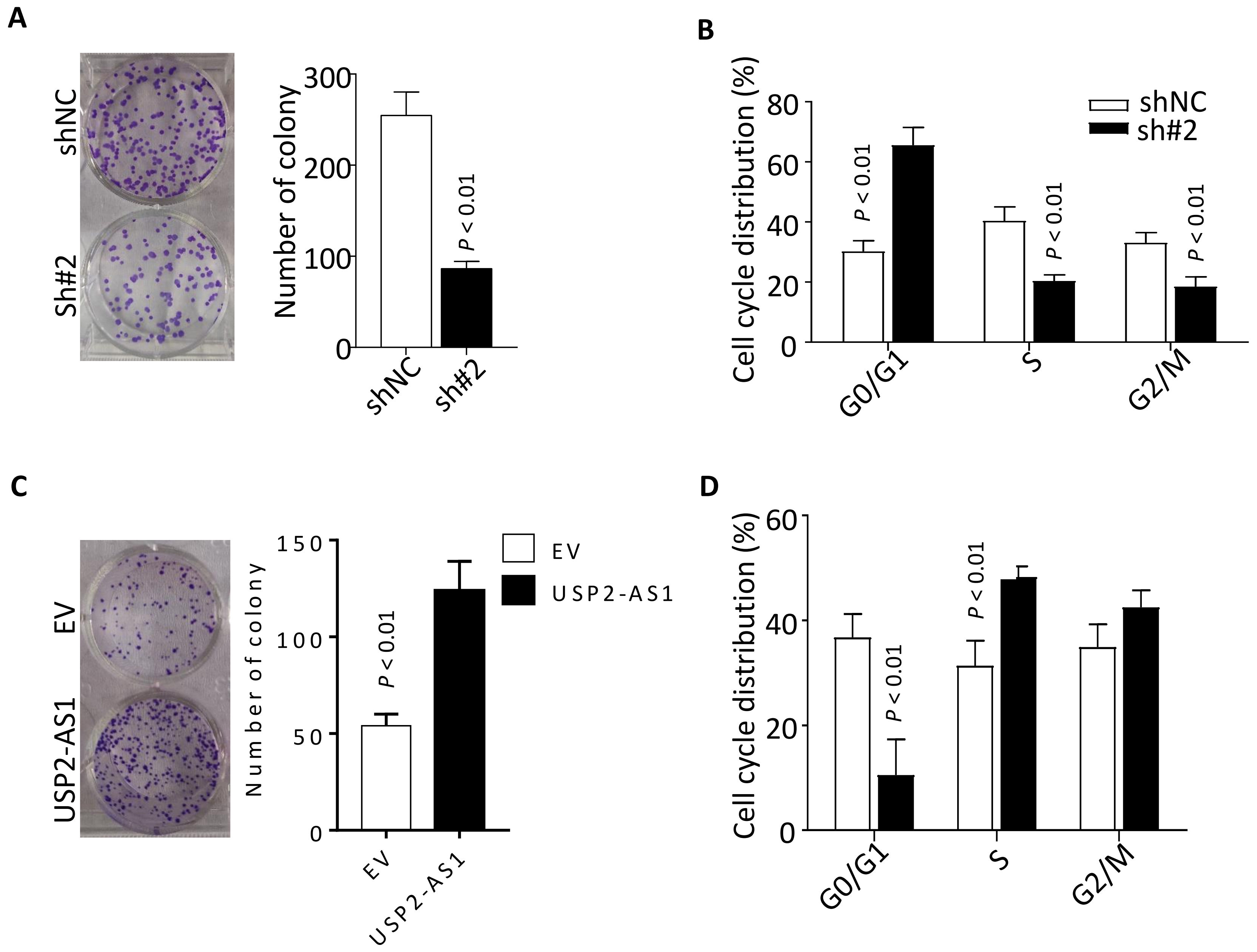

Fig. S2 USP2-AS1 promotes HNSC cells proliferation

(A) Cellular colony formation assays revealed that knockdown of USP2-AS1 inhibits FaDu cell proliferation and growth;

(B) Cell cycle analysis revealed that USP2-AS1 knockdown arrested FaDu cell cycle at G0/G1 stage;

(C) Cellular colony formation assays revealed that overexpression of USP2-AS1 promotes CAL27 cell proliferation and growth;

(D) Cell cycle analysis revealed that USP2-AS1 overexpression promotes CAL27 cell cycle transition from G0/G1 to S stage.

All of these results were triplicate, and data presented as Mean ± SD.

Fig. S3

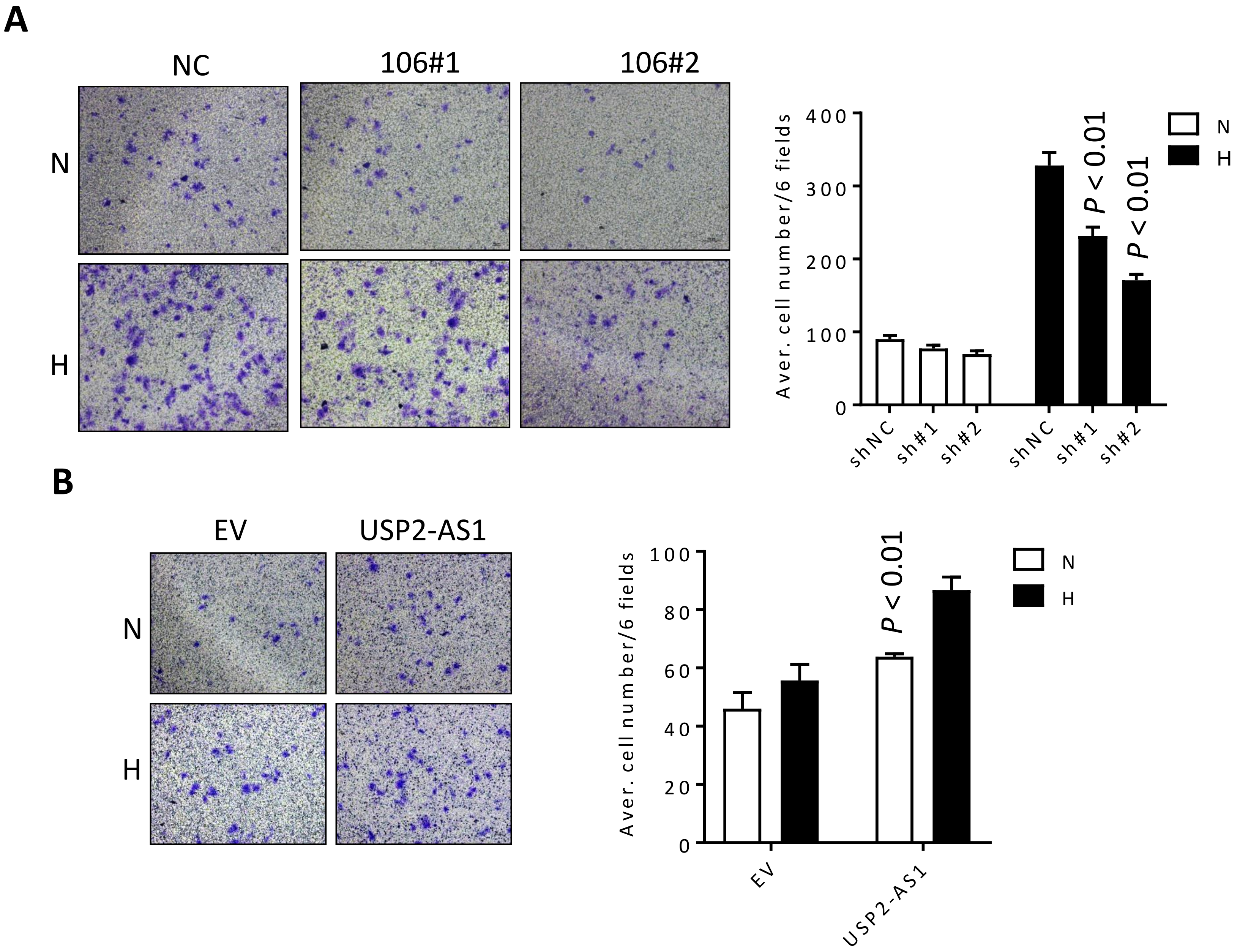

Fig. S3 USP2-AS1 promotes HNSC cells invasion under hypoxia

(A) Matrigel-coated transwell assays revealed that knockdown of USP2-AS1 inhibits FaDu cell invasion under hypoxia;

(B) Matrigel-coated transwell assays revealed that overexpression of USP2-AS1 promotes CAL27 invasion under hypoxia;

All of these results were triplicate, and data presented as Mean ± SD.

Fig. S4

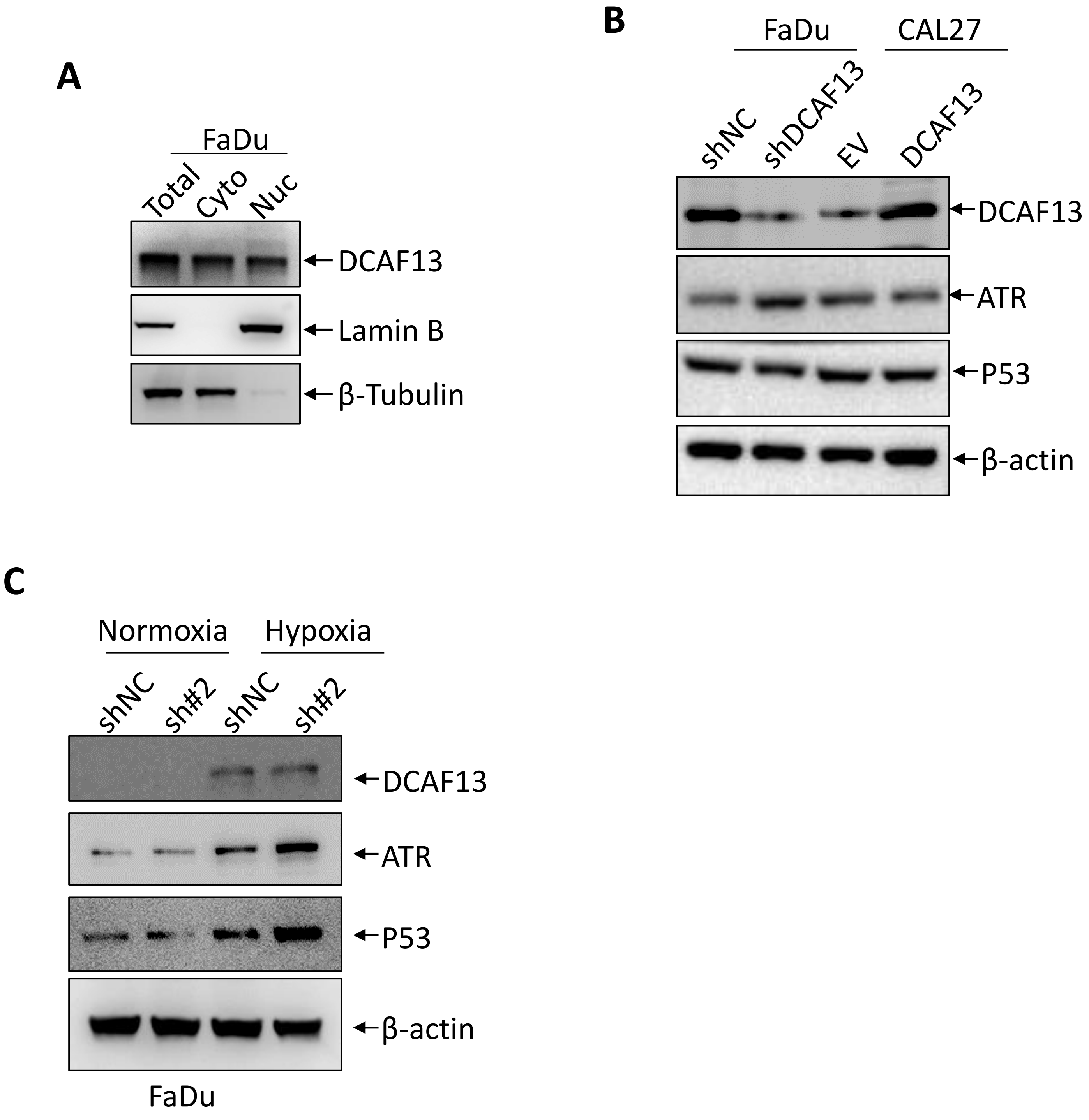

Fig. S4 DCAF13 regulates the protein level of ATR

(A) Immunoblotting analysis of indicated proteins in FaDu cell fractions. Cyto, cytoplasm; Nuc, nucleus.

(B) Immunoblotting analysis of indicated proteins in indicated FaDu and CAL27 stably transfected cells.

(C) Immunoblotting analysis of indicated proteins in indicated FaDu stably transfected cells under normoxia and hypoxia.

All of these assays were repeated twice.
