# Supplementary material for: Hypoxia-Regulated lncRNA USP2-AS1 Drives Head and Neck Squamous Cell Carcinoma Progression
Source: Cells. 2022 Oct 28;11(21):3407. doi: 10.3390/cells11213407 (PMC9655520; doi:10.3390/cells11213407)
Supplement: Supplementary file 1 [file cells-11-03407-s001.zip › cells-1912215-supplementary/suppl materials/Table S1.pdf]

Table S1 Sequences of primers and shRNAs.

Name	Sequence (5’-3’)
USP2-AS1 primer	F: GGGAGAGGCAGAGAGATCCA
	R: CAAGGGAGCAGGACAGTGAG
U1 primer	F: CTGCGTAGTTGTGCTGATGT
	R: GGCGGATTGGAAATGCTT
GAPDH primer	F: GTAACCCGTTGAACCCCATT
	R: CCATCCAATCGGTAGTAGCG
18S RNA	F: GTAACCCGTTGAACCCCATT
	R: CCATCCAATCGGTAGTAGCG
CCND1	F: GATGCCAACCTCCTCAACGA
	R: GGAAGCGGTCCAGGTAGTTC
BCL2	F: CTTTGAGTTCGGTGGGGTCA
	R: GGGCCGTACAGTTCCACAAA
BAX	F: CATGGGCTGGACATTGGACT
	R: AAAGTAGGAGAGGAGGCCGT
PGK1	F: CCACTGTGGCTTCTGGCATA
	R: ATGAGAGCTTTGGTTCCCCG
DCAF13	F: CTAGCGGACACCTCGTGGA
	R: CCAGCGAAGCAAGGAATGGT
HAS2-AS1	F: GGAACTCGACAGGACCTTGA
	R: TTGTGCGACGTGATGAAAGC
LUCAT1	F: AGGATGAGACTTAGCGTGCC
	R: CCTCGGGTTGCCTCTGTTTA
E2F1	F: GTGTAGGACGGTGAGAGCAC
	R: ATGGGGCAGAAGAACAGCTC
E2F2	F: TAGTAAACGGCAGCTGTGGG
	R: GCTTGTCTCTGCTGGACCAT
CDK2	F: GACACGCTGCTGGATGTCA
	R: CAGAAAGCTAGGCCCTGGAG
LINC00703	F: GAGGTTGAGCCCACAATCAAAC
	R: GGGACAAGTCCATCACCATGAA
PDK1	F: TGCTGTATGGCCTGCAAGAT
	R: ACATTCTGGCTGGTGACAGG
LDHA	F: TGTCTCTGGCAAAGTGGATATCTT
	R: CTGGGTGCAGAGTCTTCAGAG
H19	F: GACTCAGGAATCGGCTCTGG
	R: TGGCCATGAAGATGGAGTCG
HIF1A-AS3	F: GTACCTTCCGCATGCTTGGT

	R: ACACCCGATCTCCGTGTACTA
HIF1A-AS1	F: CTCCATGGTGAATCGGTCCC
	R: CTGGGATCCGGGAGTCAATG
ACTB	F: CATGTACGTTGCTATCCAGGC
	R: CTGGGATCCGGGAGTCAATG
Sh-#1	CCGGTTCAGCGATATGAAGTTAAATCTCGAGATTTAACTTCATATCGCTGAATTTTTG
Sh-#2	CCGGCTCAGCGAGATGAAGAGTTTACTCGAGTAAACTCTTCATCTCGCTGAGTTTTTG
Sh-#3	CCGGACAGGCTTTGGAGTCCGAGATCTCGAGATCTCGGACTCCAAAGCCTGTTTTTG
ShHIF1α#1	CCGGCCGCTGGAGACACAATCATATCTCGAGATATGATTGTGTCTCCAGCGGTTTTTG
ShHIF1α#2	CCGGCCGCTGGAGACACAATCATATCTCGAGATATGATTGTGTCTCCAGCGGTTTTTG
shMYC	CCGGCCCAAGGTAGTTATCCTTAAACTCGAGTTTAAGGATAACTACCTTGGGTTTTTG
HRE6-7	F: AGGAGGTAGGATGTTCACTCT
	R: CTGAGCGACACTGACAAACC
HRE8-9	F: CAGCCGGCTCTCTCCCTATAT
	R: CTTCGTCAGCTGGTGCTCAC
HRE10-11	F: ATTAGAGCCCGGCCTCCGGC
	R: TTCCAGCTGCCCCGTCGTGT
PDK1	F: TTAAACAGCTCACTCAAAGGC
	R: CAGCCGGCGAGGTGTGGGGTG
